# Seizure outcomes in relation to the extent of resection of the perifocal fluorodeoxyglucose and flumazenil PET abnormalities in anteromedial temporal lobectomy

**DOI:** 10.1007/s00701-015-2578-2

**Published:** 2015-09-08

**Authors:** Milo Stanišić, Christopher Coello, Jugoslav Ivanović, Arild Egge, Torsten Danfors, John Hald, Einar Heminghyt, Marjan Makki Mikkelsen, Bård Kronen Krossnes, Are Hugo Pripp, Pål Gunnar Larsson

**Affiliations:** Department of Neurosurgery Rikshospitalet, Oslo University Hospital, Sognsvannsveien 20, 0027 Oslo, Norway; PET Core Facility/PET Centre, Oslo University Hospital, Oslo, Norway; Section of Nuclear Medicine & PET, Department of Surgical Sciences, Uppsala University, Uppsala, Sweden; Department of Radiology, Oslo University Hospital, Oslo, Norway; Department of Clinical Psychology and Neuropsychology, National Center for Epilepsy, Oslo University Hospital, Oslo, Norway; Department of Adult Epilepsy, National Center for Epilepsy, Oslo University Hospital, Oslo, Norway; Department of Pathology, Oslo University Hospital, Oslo, Norway; Oslo Center of Biostatistics and Epidemiology, Research Support Service, Oslo University Hospital, Oslo, Norway; Clinical Neurophysiologic Laboratories, Department of Neurosurgery, Oslo University Hospital, Oslo, Norway

**Keywords:** Temporal lobe epilepsy, ^18^F-FDG-PET, ^11^C-FMZ-PET, Post-operative outcome

## Abstract

**Background:**

The area of predominant perifocal [^18^F]fluorodeoxyglucose (^18^F-FDG) hypometabolism and reduced [^11^C]flumazenil (^11^C-FMZ) -binding on PET scans is currently considered to contain the epileptogenic zone and corresponds anatomically to the area localizing epileptogenicity in patients with temporal lobe epilepsy (TLE). The question is whether the volume of the perifocal pre-operative PET abnormalities, the extent of their resection, and the volume of the non-resected abnormalities affects the post-operative seizure outcome.

**Methods:**

The sample group consisted of 32 patients with mesial temporal sclerosis who underwent anteromedial temporal lobe resection for refractory TLE. All patients had pathologic perifocal findings on both of the PET modalities as well as on the whole-brain MRI. The volumetric data of the PET and MRI abnormalities within the resected temporal lobe were estimated by automated quantitative voxel-based analysis. The obtained volumetric data were investigated in relation to the outcome subgroups of patients (Engel classification) determined at the 2-year post-operative follow-up.

**Results:**

The mean volume of the pre-operative perifocal ^18^F-FDG- and ^11^C-FMZ PET abnormalities in the volumes of interest (VOI) of the epileptogenic temporal lobe, the mean resected volume of these PET abnormalities, the mean volume of the non-resected PET abnormalities, and the mean MRI-derived resected volume were not significantly related to the outcome subgroups and had a low prediction for individual freedom from seizures.

**Conclusions:**

The extent of pre-surgical perifocal PET abnormalities, the extent of their resection, and the extent of non-resected abnormalities were not useful predictors of individual freedom from seizures in patients with TLE.

## Introduction

Temporal lobe epilepsy (TLE) is the most common partial seizure syndrome in adults. TLE is, unfortunately, often refractory to optimal pharmacological treatment [44]. Evidence suggests that surgical treatment offers the possibility of freedom from seizures for these patients [10]. However, it is unknown why a relatively large proportion of patients continue to experience seizures after surgery despite careful pre-surgical work-up and selection. Furthermore, the predictors for individual freedom from seizures in patients with TLE are not fully understood.

The common view is that an accurate pre-surgical lateralization and localization of epileptogenic zone in patients with drug-resistant TLE is essential for postoperative seizure freedom. However, identifying the epileptogenic zone can be complex and may require a constellation of findings from multimodal evaluation methods. Interictal [^18^F]fluorodeoxyglucose (^18^F-FDG) PET, an index of the cerebral metabolism rate of glucose, and interictal [^11^C]flumazenil-binding (^11^C-FMZ) PET, an index of the binding potential of brain benzodiazepine receptors, are widely used as helpful functional neuroimaging tools of non-invasive localization and characterization of epileptogenic regions [17, 18, 25, 26, 29, 30, 49, 55]. Because it has been shown that the interictal ^18^F-FDG PET usually shows a large area of reduced radiotracer uptake (hypometabolism) extending beyond the epileptogenic zone [1, 24] and the interictal ^11^C-FMZ PET may show a more restricted area of decreased tracer binding [31, 40] in patients with refractory TLE, these neuroimaging modalities can be used for lateralization and general localization of the seizure focus, making an a priori hypothesis about subsequent intracranial electrode placement possible [28]. However, research on the relationship between the extent of the resection of PET abnormalities and outcomes in patients with refractory TLE has been less of focus [53].

The area of predominant glucose hypometabolism in the temporal lobe is currently considered to contain the epileptogenic zone [33, 51] and corresponds anatomically to the area localizing epileptogenicity [45]. Furthermore, it is assumed that the area of localized reduction of ^11^C-FMZ binding correlates closely with the side and site of seizure onset [38, 47]. Therefore, the question is whether the volume of the perifocal pre-operative PET abnormalities, the extent of their resection, and the volume of the non-resected abnormalities affects the post-operative seizure outcome. This study was designed to investigate the relationship between these volumetric data of ^18^F-FDG- and ^11^C-FMZ PET abnormalities that occurred in the epileptogenic temporal lobe and were accessible for resection and the post-surgical seizure outcome of anteromedial temporal lobectomy (AMTL) in the TLE patients with mesial temporal sclerosis. We also quantitatively determined the magnetic resonance imaging (MRI)-derived resected volumes of the temporal lobe and analyzed whether this value was associated with post-operative seizure outcome. It should be noted that this study was not designed to indicate the feasibility of ^18^F-FDG- and ^11^C-FMZ PET investigations in pre-surgical work-up.

## Materials and methods

The study was approved by the Regional Ethical Committee of Health Region in South-East Norway (2012/1989/REK) for the study of human subjects.

### Patients

Between July 2002 and April 2008 at the Department of Neurosurgery at Oslo University Hospital, Norway, 107 patients with medically refractory TLE underwent unilateral AMTL. Among these patients, 39 with a previous temporal lobe surgery or presence of a tumor, vascular lesion, cortical developmental anomaly, and encephalomalacia were excluded for this study. We identified 68 patients in which MRI and histopathologic examinations revealed hippocampal sclerosis. Thirty-six patients of these 68 were not included in the study because they did not fulfil the required inclusion criteria. Determination of the presence of PET abnormalities was performed by visual inspection and a semi- quantitative method of interpretation with the interpreter comparing any hemispheric asymmetry with a normal ^18^F-FDG- and ^11^C-FMZ PET brain pattern. Patients with bilateral temporal lobe PET abnormalities, based on this evaluation, were not included in this cohort because the implemented automated method of PET image analysis detailed below precludes the detection of bilateral PET abnormalities in homologous temporal lobe regions. The rest, 32 patients, who had ^18^F-FDG- and ^11^C-FMZ PET neuroimaging performed as part of the pre-surgical evaluation, pre- and post-surgical high-resolution whole-brain MRI, and post-operative seizure outcome determined at the 2-year post-operative follow-up were included in this study.

Pre-surgical evaluation included seizure history and semiology, neurological examination, long-term video EEG monitoring, brain MRI, neuropsychological examinations, and interictal ^18^F-FDG- and ^11^C-FMZ PET imaging. When appropriate, ictal and interictal brain single-photon emission computed tomography (SPECT) was also performed. On the basis of these multimodal investigations, the diagnosis of TLE was confirmed, and localizing information was reviewed at the pre-surgical planning meeting.

### EEG protocol and assessment

Pre-operatively, long-term interictal and ictal scalp EEGs were recorded by a video EEG monitoring system in all patients, with 25 or 64 electrodes placed according to the international 10–10 system. In addition, 56 % (18 out of 32) patients had long-term intracranial EEG and video recordings with surgically placed subdural strip electrodes (including all patients with discordant localization information from other modalities of evaluation) to confirm the epileptogenic zone before undergoing the temporal lobe resection. The subdural electrode placements were guided by seizure semiology, probable seizure onset area as determined by scalp interictal and ictal EEGs, and ^18^F-FDG- and ^11^C-FMZ PET abnormalities. Multiple ictal recordings were obtained during seizures typical for the patient and the temporal lobe of seizure onset was defined. The seizure onset temporal lobe and seizure onset zone were identified by identifying seizure onset, early seizure spread (defined as areas involved in seizure activity less than 10 s after onset of the seizure) and dominant interictal epileptiform activity consisting of sharp and slow wave components.

### MRI acquisition and qualitative visual assessment

Pre-surgical high-resolution whole-brain MRI examinations were carried out using a TLE protocol on a 1.5-Tesla scanner (Avanto, Siemens, Erlangen, Germany) with a 12-channel headcoil. The following sequences were obtained: 3D T1-weighted MP RAGE (TR/TE 1900/3.46), flip angle of 15 degrees, slice thickness 1.25 mm, and FOV 250; coronal FLAIR (TR/TI/TE 9000/2500/108) slice thickness 3 mm, FOV 230, and axial T2-weighted (TR/TE 4050/98) slice thickness 5 mm, FOV 230. Pre-operatively, all MR images were reviewed by a neuroradiologist with specific expertise in epilepsy studies using accepted criteria. The presence of hippocampal sclerosis on MRI scans (predominant unilateral atrophy and increased T2 signal in the hippocampus) were determined. Similar sequences were also obtained post-operatively, 6 months after surgery, to confirm the extent of the AMTL.

Qualitative visual radiological assessment of the pre-surgical MRI studies in these patients demonstrated mesial temporal sclerosis localized to the resected temporal lobe in all included patients (Table 1).

**Table 1:** Demographic and clinical profile of 32 patients with drug-resistant complex partial seizures of temporal lobe origin with or without secondary generalization

Characteristic	*n* (%) or median (min-max)
Gender	
Female	21 (66 %)
Male	11 (34 %)
Etiological	
Febrile convulsion	8 (25 %)
Meningitis	2 (6 %)
Unknown	22 (69 %)
Age onset (years)	
Median (min-max)	7 (1–34)
Duration of epilepsy before surgery (years)	
Median (min-max)	24 (6–54)
Age Surgery (years)	
Median (min-max)	35 (10–55)
MRI results	
Structural finding localized to the temporal lobe	32 (100 %)
EEG seizure onset defined by	
Scalp EEG	14 (44 %)
Intracranial EEG	18 (56 %)
Resected temporal lobe	
Left	20 (62 %)
Right	12 (38 %)
Histopathological findings in hippocampus/neocortex	
Hippocampal sclerosis/no pathologic*	32 (100 %)
Seizure outcome score at 2-year follow-up (Engel’s class)	
Class I	25 (78 %)
Class II	1 (3 %)
Class III	2 (6 %)
Class IV	4 (13 %)

Data presented as number of patients (%) or median (min-max)

*The specimens with hippocampal sclerosis are considered pathologic, whereas the specimens with gliosis only are considered as not pathologic findings

### ^18^F-FDG- and ^11^C-FMZ PET acquisition and pre-surgical evaluation

Interictal (no seizures within the last 24 h) out-patient ^18^F-FDG- and ^11^C-FMZ PET scans were acquired at the University Hospital in Uppsala, Sweden. The patients underwent a 40-min dynamic scan after intravenous administration of 4 MBq/kg ^11^C-FMZ and a 20-min static scan 30 min after the i.v. administration of 3 MBq/kg ^18^F-FDG on the same day. The images were acquired using an ECAT Exact HR+scanner (Siemens/CTI, Knoxville, TN, USA) and reconstructed using normalization and attenuation-weighted ordered subsets expectation maximization (six iterations and eight subsets) after applying all appropriate corrections. The reconstruction process created a standard series of contiguous images oriented in the transaxial, coronal, sagittal, and transtemporal planes. Evaluation and reporting of imaging data were performed by experienced nuclear medicine physicians. Images were evaluated visually and with a semi-quantitative method with measurements of inter-hemispheric asymmetries. All significant findings were reported without or with limited access to the previous pre-surgical investigation. Routine EEG monitoring to confirm that no seizure occurred during acquisition was not performed.

This evaluation of the pre-surgical ^18^F-FDG -and ^11^C-FMZ PET studies in these patients revealed that 31 patients (97 %) had decreased glucose metabolism and 26 patients (81 %) had decreased ^11^C-FMZ binding that occurred in the temporal lobe of seizure onset in accordance with the EEG results, which are considered perifocal PET abnormalities in this study. In addition, remote PET abnormalities that extended either contiguously beyond the resected temporal lobe (into the adjacent lobe) or presented as remote ipsilateral and/or contralateral PET abnormalities (outside of the resected lobe and not continuous with the resected temporal lobe) were found in 21 patients (66 %) on ^18^F-FDG and 18 (56 %) on ^11^C-FMZ PET. Such remote PET abnormalities were not included in neuroimaging post-processing and quantitative analyses in this study.

### Surgery, histology, and postoperative seizure outcome

All patients subsequently underwent an AMTL under general anesthesia. Five of them, in which pre-surgical neuropsychometric memory tests were not affected, had undergone a tailored AMTL of the dominant hemisphere. One of the four neurosurgeons using similar microsurgical resection techniques performed the surgical procedures. The anterior temporal lobe was removed, with an en bloc excision of neocortical structures that were submitted to histopathological examination, followed by resection of the amygdala, and subsequent en bloc resection of the parahippocampal gyrus and hippocampus that were also submitted to histopathological examination. The extent of lateral neocortical resection along the long axis of the temporal lobe included the superior temporal gyrus limited to approximately 2 cm from the temporal tip and the middle, inferior, and fusiform temporal gyri 3.5 to 5 cm from the temporal tip as measured at the time of surgery. The medial temporal lobe resection included endopial emptying of the uncus and en bloc resection of the parahippocampal gyrus medially to the tentorial incisura and the hippocampus with fimbria followed by generous resection of the amygdala. The hippocampus resection extended posteriorly to the posterior margin of the midbrain and exposure of the anterior portion of the ventricular atrium (approximately 2.5 to 3 cm from the pes hippocampi anterior tip in the temporal horn posteriorly along its length). The margins of the AMTL were not based on the extent of ^18^F-FDG- and ^11^C-FMZ PET abnormalities unless there was corresponding epileptiform EEG abnormality occurring over the temporal lobe.

The resected specimens were histopathologically examined by a neuropathology expert using a standard protocol for epilepsy surgery cases. In brief, surgical specimens from the neocortex and hippocampus were fixed in formalin and sectioned perpendicularly to the cortical surface into several tissue blocks. All the tissue blocks from the neocortex and the hippocampus were embedded in paraffin and stained with hematoxylin and eosin (H&E) and Luxol fast blue. Various immunostains (such as GFAP, myelin basic protein, phosphorylated neurofilament protein, NeuN, synaptophysin, and αB-crystallin) were applied where necessary to clarify the nature of the pathology. The result of the histopathological examination is presented in Table 1.

Postoperative seizure outcome at the 2-year follow-up (Table 1) was determined by experienced neuropsychology experts according to the Engel classification [11] . For analysis in this cohort, seizure outcome data were dichotomized into Engel class I outcome and Engel class II–IV outcome.

### Neuroimaging post-processing and quantitative automated voxel-based analyses

**Fig. 1:**
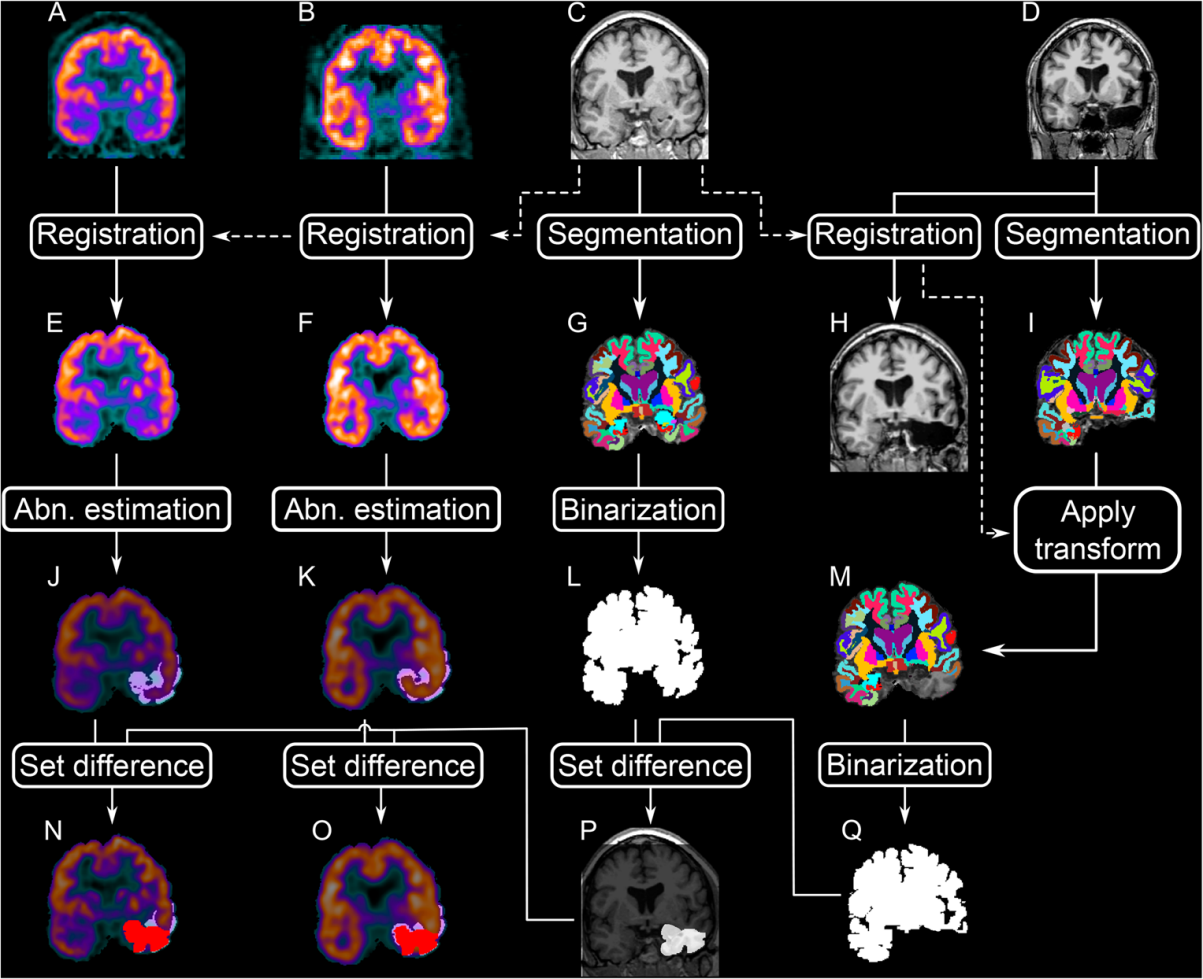
Schematic of the post-processing of the MR and PET images. **a** Raw static ^18^F-FDG. **b** Raw static ^11^C-FMZ. **c** Pre-operative MRI. **d** Post-operative MRI. **e** ^11^F-FDG image co-registered to the pre-operative MRI. **f** ^11^C-FMZ image co-registered to the post-operative MRI. **g** Labeled (or segmented) pre-operative MRI. **h** Post-operative MRI co-registered to the pre-operative MRI. **i** Labeled (or segmented) post-operative MRI. **j** Temporal lobe ^18^F-FDG abnormality overlaid on the co-registered ^18^F-FDG image. **k** Temporal lobe ^11^C-FMZ abnormality overlaid on the co-registered ^11^C-FMZ image. **l** Binary map of the skull-stripped pre-operative MRI. **m** Labeled (or segmented) post-operative MRI co-registered to the pre-operative MRI. **n** Resected area overlaid on the temporal lobe ^18^F-FDG abnormality overlaid on the co-registered ^18^F-FDG image. **o** Resected area overlaid on the temporal lobe ^11^C-FMZ abnormality overlaid on the co-registered 11C-FMZ image. **p** Estimated resected area. **q** Binary map of the skull-stripped co-registered post-operative MRI

The steps involved in the post-processing of the images are summarized in Fig. 1. All post-acquisition imaging processing and analyses were carried out by a single operator blinded to the post-surgical seizure outcome. T1-weighted MPRAGE MR image slices in DICOM format were converted into a single 3D volume and the PET images in ECAT format were converted into a single 3D volume. The initial volume dimensions of the T1-weighted MPRAGE MR volume were 512 × 512 × 160 with a voxel size of 0.49 × 0.49 × 1.25 mm^3^. The initial volume dimension of ^18^F-FDG PET and ^11^C-FMZ PET was 128 × 128 × 63 with a voxel size of 2.06 × 2.06 × 2.43 mm^3^.

#### Estimation of resection volume extent in the temporal lobe of seizure onset using pre- and post-surgical MRI

Pre- and post-surgical high-resolution MR volumes (Fig. 1c and d) were segmented using Freesurfer 5.1.0 [13, 14]. The surface-based segmentation tool estimates the region to which each voxel of the input MRI most probably belongs, generates as output a label volume containing one label (region) per voxel (Fig. 1g and i) and calculates the cortical thickness and volume for each label. In the same process, the original MRI volumes were re-sliced to an isotropic voxel size (1 mm^3^).

The resected volume in the temporal lobe of seizure onset was automatically calculated by the following procedure. The post-surgical volume was registered using a rigid registration approach [36] (Fig. 1h) and the calculated transformation matrix was applied to the post-surgical labels (Fig. 1m). Both pre- and post-surgical labels were converted to binary volumes and morphological operations (closing) were applied to fill unassigned voxels (Fig. 1l and q). After this step, voxel-by-voxel subtraction between pre- and post-surgical MRI binary volumes (equivalent to a set difference of post-surgical and pre-surgical volume) was performed (Fig. 1p). This difference corresponded to the volumetric resected area in the temporal lobe of seizure onset, which was more accurately obtained after morphological opening to remove non-resected areas that appeared in the resection volume due to co-registration error.

#### Definition of volumes of interests using pre- and post-surgical MRI

Two volumes of interest (VOIs) on the pre-surgical MRI binary image were automatically defined in the right and left temporal lobe as follows: (1) the medial gathering of the amygdala, hippocampus, and parahippocampal gyrus (VOI_A+H+PH_) and (2) the whole temporal lobe consisting of neocortex of the superior temporal, middle temporal, inferior temporal, fusiform, temporal pole, transverse temporal cortical areas, entorhinal parahippocampal, hippocampus, and amygdala structures (VOI_TL_). When cortical areas were included in the VOI, both the cortical ribbon and subcortical white matter (within 5 mm from the cortical ribbon) were included in the definition of the VOI.

Pre-surgical volumes of these two VOIs were calculated by summing the individual volumes extracted from the pre-surgical MR label volume (Fig. 1g). Calculations of the post-surgical volumes of VOIs in the resected temporal lobe were performed with a different method because many assumptions of Freesurfer algorithms would be violated by resected MR images. To overcome this problem, a derived post-surgical label volume was estimated by voxel-by-voxel subtraction of the pre-surgical label volume (Fig. 1g) and the estimated resected volume (resVol) (Fig. 1p). Using this derived post-surgical MR label volume, the post-surgical volumes of these two VOIs in the temporal lobe of seizure onset were calculated by summing the individual volumes extracted from the derived post-surgical MR label volume.

#### Estimation of pre-surgical ^18^F-FDG hypometabolism and decreased ^11^C-FMZ binding in the VOIs of the epileptogenic temporal lobe

Dynamic ^11^C-FMZ was averaged between the tenth and 20th minutes of the experiment to create a static ^11^C-FMZ volume. Both the static ^18^F-FDG (Fig. 1a) and ^11^C-FMZ (Fig. 1n) volumes were rigidly co-registered to the pre-surgical MRI using a boundary-based registration algorithm [15] (Fig. 1e and f) and re-sliced in the pre-surgical space. At this stage, the pre-surgical MRI label volume of VOIs, volumetric resected area of VOIs and ^18^F-FDG- and ^11^C-FMZ PET images were in the same space, allowing voxel-by-voxel operations between these volumes.

**Fig. 2:**
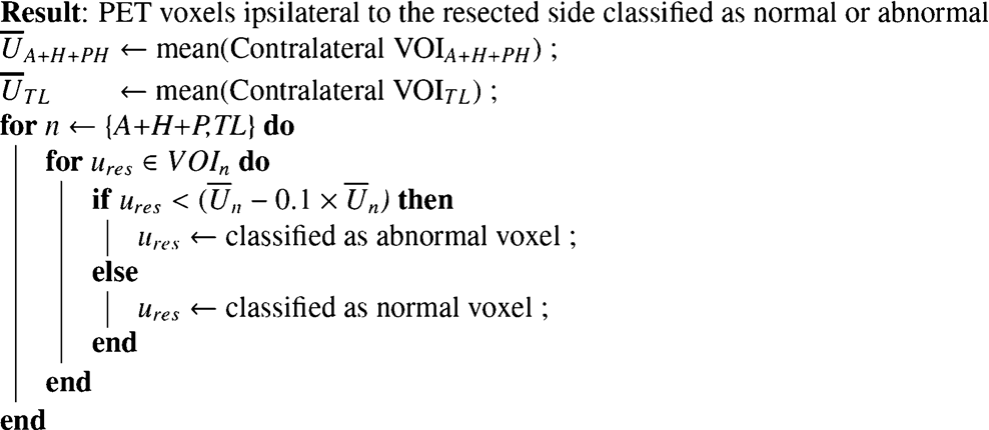
Algorithm 1: Classification of PET voxels ipsilateral to the resected side

The ^18^F-FDG-and ^11^C-FMZ PET abnormality areas that lay within the VOIs of epileptogenic temporal lobe ipsilateral to the resected side were defined by the following voxel-based procedure. The mean uptake of both tracers in each defined VOI (VOI_A+H+PH_ or VOI_TL_) was calculated contralateral from the resected side (Ū_A+H+PH_ or Ū_TL_). Each voxel of the VOI on the epileptogenic resected side (*u*_res_) were then compared to the contralateral mean and voxels with uptake 10 % less than the contralateral mean were defined as marked “perifocal” PET abnormalities that occurred in the temporal lobe on resected side (see Fig. 2. Algorithm 1). As an output of this algorithm, four pre-surgical abnormality volumes (PreAbnVol) were created in the epileptogenic temporal lobe, one per tracer and per VOI (PreAbnVol^18^F-FDG_A+H+PH_, PreAbnVol^18^F-FDG_TL_, PreAbnVol^11^C-FMZ_A+H+PH_, and PreAbnVol^11^C-FMZ_TL_). The definition of abnormalities was implemented using Matlab 2009a (The MathWorks, Inc., Natick, MA, USA). This procedure allowed the definition of pre-surgical perifocal ^18^F-FDG- and ^11^C-FMZ PET abnormalities in the VOIs of the epileptogenic temporal lobe based on an asymmetry index derived from contralateral homotopic VOIs in the temporal lobe.

#### Estimation of non-resected volume of ^18^F-FDG- and ^11^C-FMZ PET abnormalities in VOIs using voxel-by-voxel comparison

The extent of the ^18^F-FDG and ^11^C-FMZ abnormalities was calculated by counting the number of abnormal voxels in each pre-surgical PET abnormality volume. The extent of non-resected perifocal ^18^F-FDG and ^11^C-FMZ abnormality volumes in VOIs was calculated as the relative complements (or set difference) between the abnormality volumes and the resection volume using the following algorithm:

$$ \mathrm{nonResAbnVol}=\mathrm{abnVol}\backslash \mathrm{resVol}=\left\{\left.\chi \in \mathrm{abnVol}\right|\chi \notin \mathrm{resVol}\right\} $$

As an output of this algorithm, four non-resected abnormality volumes (nonResAbnVol) were created in the epileptogenic temporal lobe, one per tracer and per VOI (nonResAbnVol^18^F-FDG_A+H+PH_, nonResAbnVol^18^F-FDG_TL_, nonResAbnVol^11^C-FMZ_A+H+PH_, and nonResAbnVol^11^C-FMZ_TL_). The non-resected volumes of the ^18^F-FDG- and ^11^C-FMZ PET abnormalities were expressed as a percentage of the total marked PET abnormality volume in VOIs.

### Statistical analysis

All statistical analyses were performed with SPSS, version 21.0, (IBM Corporation, Armonk, NY, USA). The data were described as number and percentage or mean and standard deviation unless stated otherwise. The difference in volumes between perifocal ^18^F-FDG PET and ^11^C-FMZ PET abnormalities in the corresponding VOIs were statistically analyzed using a paired-samples *t* test. Patients with a satisfactory (Engel class I) and unsatisfactory (Engel classes II–IV) post-surgical outcome were compared using an independent samples *t* test for the following five variables: (i) the pre-surgical volume of the present perifocal ^18^F-FDG hypometabolism and decreased ^11^C-FMZ binding in the VOI_A+H+PH_ and VOI_TL_; (ii) the volume of the resected perifocal ^18^F-FDG hypometabolism and decreased ^11^C-FMZ binding that lay in the VOI_A+H+PH_ and VOI_TL_; (iii) the percentage of the non-resected perifocal ^18^F-FDG hypometabolism and decreased ^11^C-FMZ binding that present in the VOI_A+H+PH_ and VOI_TL_ after resection; (iv) the MRI-derived resected tissue volume in the VOI_A+H+PH_ and VOI_TL_; (v) the percentage represented by the resected MRI-defined volume within the VOI_A+H+PH_ and VOI_TL_.

To assess the possible influence of all five variables on the achievement of a satisfactory (Engel class I) post-surgical seizure outcome, we analyzed the data with logistic regression and estimated odds ratio (OR) and corresponding C-statistic (equivalent to the area under the receiver operating characteristics curve). For all tests, *p* < 0.05 was considered to be a statistically significant difference between the groups.

## Results

The cohort consisted of 32 patients (21 female and 11 male) with a median age of 35 years (min 10–max 55). At the latest follow-up (2 years after surgery), 78 % of patients (25 out of 32) had an Engel class I outcome (i.e., free of disabling seizures) of whom 56 % (18 out of 32) had Engel class IA outcome (i.e., completely seizure free) and 22 % (seven out of 32) Engel class IB outcome (i.e., nondisabling simple partial seizures only). Seven (22 %) of 32 patients had unsatisfactory post-surgical outcome (Engel class II–IV). The baseline demographic and clinical features of the patients are summarized in Table 1.

### The results of the automated quantitative ^18^F-FDG- and ^11^C-FMZ PET analysis

The quantitative voxel-based evaluation of the pre-surgical ^18^F-FDG- and ^11^C-FMZ PET studies in the patients revealed that all 32 patients (100 %) had perifocal ^18^F-FDG hypometabolism and decreased ^11^C-FMZ binding in the epileptogenic temporal lobe ipsilateral to the resected side. In the study, the mean pre-surgical perifocal volume of the ^18^F-FDG PET abnormality in the VOI_A+H+PH_ was not significantly different from the ^11^C-FMZ PET abnormality volume (*p* = 0.178). However, in the VOI_TL_, the mean pre-surgical perifocal volume of the ^18^F-FDG PET abnormality was significantly larger than the ^11^C-FMZ PET abnormality volume (*p* < 0.001).

In the VOI_A+H+PH_, the mean resected volume of the perifocal ^18^F-FDG PET abnormality was significantly higher than the resected volume of the perifocal ^11^C-FMZ PET abnormality (*p* = 0.005). Similarly, the mean resected volume of the perifocal ^18^F-FDG PET abnormality in the VOI_TL_ was significantly higher than the mean resected volume of the perifocal ^11^C-FMZ PET abnormality (*p* < 0.001).

In the VOI_A+H+PH_, the percentage of the resected volume of the perifocal ^18^F-FDG PET abnormality of 19 % was significantly higher than the percentage of the resected volume of the perifocal ^11^C-FMZ PET abnormality of 14 % (*p* < 0.001). Similarly, in the VOI_TL,_ the percentage of the resected volume of the perifocal ^18^F-FDG PET abnormality of 43 % was significantly higher than the percentage of the resected volume of the perifocal ^11^C-FMZ PET abnormality of 37 % (*p* < 0.001).

Following resection, in the Engel class I and Engel class II–IV outcome subgroups of patients, the mean pre-surgical volumes of perifocal ^18^F-FDG-and ^11^C-FMZ PET abnormalities were statistically significantly reduced in the VOI_A+H+PH_ (Table 2) and the VOI_TL_ (Table 3).

**Table 2:** Comparisons of the mean pre-surgical and post-surgical volumes of perifocal ^18^F-FDG PET and ^11^C-FMZ PET abnormalities in the VOI_A+H+PH_ and the mean resected volumes of abnormalities in relation to seizure outcome subgroups

^18^F-FDG-and ^11^C-FMZ PET in VOI_A+H+PH_	Seizure outcome scores (Engel class)	*n*	Volume of abnormality mean (SD) (mm^3^)	Volume of resected abnormality mean (SD) (mm^3^)	95 % CI	*p* value
^18^F-FDG						
Before resection	Class I	25	3221 (1537)	712 (1053)	277–1146	0.002
After resection			2509 (868)			
Before resection	Class II–IV	7	3007 (1344)	521 (367)	181–860	0.009
After resection			2486 (1114)			
^11^C-FMZ						
Before resection	Class I	25	3101 (617)	428 (435)	248–608	<0.001
After resection			2673 (574)			
Before resection	Class II–IV	7	2685 (691)	447 (369)	106–788	0.018
After resection			2238 (585)			

*VOI* volume of interest, _*A+H+PH*_ Amygdala+Hippocampus+Parahippocampal gyrus, *N* number of patients

**Table 3:** Comparisons of the mean pre-surgical and post-surgical volumes of perifocal ^18^F-FDG PET and ^11^C-FMZ PET abnormalities in the VOI_TL_ and the mean resected volumes of abnormalities in relation to seizure outcome subgroups

^18^F-FDG-and ^11^C-FMZ PET in VOI_TL_	Seizure outcome scores (Engel class)	*n*	Volume of abnormality mean (SD) (mm^3^)	Volume of resected abnormality mean (SD) (mm^3^)	95 % CI	*p* value
^18^F-FDG						
Before resection	Class I	25	33,358 (7224)	14,461 (4751)	12,500–16,422	<0.001
After resection			18,896 (5670)			
Before resection	Class II–IV	7	27,460 (4738)	12,518 (4848)	8035–17,001	<0.001
After resection			14,942 (3761)			
^11^C-FMZ						
Before resection	Class I	25	25,469 (6175)	9203 (3225)	7871–10,534	<0.001
After resection			16,266 (4966)			
Before resection	Class II–IV	7	22,609 (3985)	8952 (3575)	5646–12,259	<0.001
After resection			13,657 (3475)			

*VOI* volume of interest, _*TL*_ whole temporal lobe, *n* number of patients

However, the volume of perifocal ^18^F-FDG PET and ^11^C-FMZ PET abnormalities in the VOI_A+H+PH_ before resection, the resected abnormality volume, and the percentage of non-resected abnormality volumes were not significantly different in the Engel class I outcome subgroup compared with the Engel classes II–IV outcome subgroup (Table 4).

**Table 4:** Comparisons of the mean pre-surgical volumes of perifocal ^18^F-FDG- and ^11^C-FMZ PET abnormalities in the VOI_A+H+PH_, resected volumes of perifocal PET abnormalities, percentages of non-resected perifocal PET abnormalities, and the MRI-derived mean resected volume and percentage this volume represents between seizure outcome subgroups Engel class I vs. classes II–IV

^18^F-FDG-and ^11^C-FMZ PET and MRI in VOI_A+H+PH_	Seizure outcome scores (Engel class)	*n*	Volume of abnormality mean (SD) (mm^3^ or %)	Differences mean (mm^3^ or %)	95 % CI	*p* value
^18^F-FDG pre-surgical volume	Class I	25	3221 (1537)	214	−1096–1525	0.740
	Class II–IV	7	3007 (1344)			
^18^F-FDG resected volume	Class I	25	712 (1053)	191	−644–1026	0.643
	Class II–IV	7	521 (367)			
^18^F-FDG percentage of non-resected abnormality	Class I	25	82 (14)	1	−11–13	0.875
	Class II–IV	7	81 (11)			
^11^C-FMZ pre-surgical volume	Class I	25	3101 (618)	416	−137–969	0.135
	Class II–IV	7	2685 (691)			
^11^C-FMZ resected volume	Class I	25	428 (436)	−19	−388–350	0.917
	Class II–IV	7	447 (369)			
^11^C-FMZ percentage of non-resected abnormality	Class I	25	87 (12)	3	−7–13	0.535
	Class II–IV	7	84 (10)			
MRI-derived volume resected	Class I	25	1572 (1316)	63	−1000–1126	0.905
	Class II–IV	7	1509 (693)			
MRI percentage of resected volume	Class I	25	19 (15)	−2	−14–11	0.781
	Class II–IV	7	20 (10)			

*VOI* volume of interest, _*A+H+PH*_ Amygdala+Hippocampus+Parahippocampal gyrus and uncus, *n* number of patients

The volume of perifocal ^18^F-FDG PET abnormality in the VOI_TL_ before resection was significantly higher in the Engel class I outcome subgroup compared with the Engel classes II–IV outcome subgroup (Table 5). On the other hand, the volume of ^11^C-FMZ PET abnormality in the VOI_TL_ before resection was not significantly different in the Engel class I outcome subgroup compared with the Engel classes II–IV outcome subgroup (Table 5). However, the resected volume of perifocal ^18^F-FDG PET and ^11^C-FMZ PET abnormalities in the VOI_TL_ and the percentage of non-resected abnormality volumes were not significantly different in the Engel class I outcome subgroup compared with the Engel classes II–IV outcome subgroup (Table 5).

**Table 5:** Comparisons of the mean pre-surgical volumes of perifocal ^18^F-FDG- and ^11^C-FMZ PET abnormalities in the VOI_TL_ , resected volumes of perifocal PET abnormalities, percentages of non-resected perifocal PET abnormalities and the MRI-derived mean resected volume and percentage this volume represents between seizure outcome subgroups Engel class I vs. class II–IV

^18^F-FDG-and ^11^C-FMZ PET and MRI in VOI_TL_	Seizure outcome scores (Engel class)	*n*	Volumes of abnormality mean (SD) (mm^3^ or %)	Differences mean (mm^3^ or %)	95 % CI	*p* value
^18^F-FDG pre-surgical volume	Class I	25	33,358 (7224)	5898	−41–11,836	0.052
	Class II–IV	7	27,460 (4738)			
^18^F-FDG resected volume	Class I	25	14,462 (4751)	1943	−2223–6109	0.348
	Class II–IV	7	12,518 (4848)			
^18^F-FDG percentage of non-resected abnormality	Class I	25	57 (11)	2	−8–12	0.724
	Class II–IV	7	55 (11)			
^11^C-FMZ pre-surgical volume	Class I	25	25,469 (6175)	2859	−2209–7928	0.258
	Class II–IV	7	22,610 (3985)			
^11^C-FMZ resected volume	Class I	25	9203 (3226)	250	−2630–3131	0.860
	Class II–IV	7	8953 (3575)			
^11^C-FMZ percentage of non-resected abnormality	Class I	25	64 (10)	3	−6–12	0.503
	Class II–IV	7	61 (11)			
MRI-derived volume resected	Class I	25	19,647 (5782)	1650	−3426–6725	0.512
	Class II–IV	7	17,997 (5928)			
MRI percentage of resected volume	Class I	25	27 (8)	0	−7–7	0.941
	Class II–IV	7	27 (8)			

*VOI* volume of interest, _*TL*_ whole temporal lobe, *n* number of patients

The odds ratio from logistic regression of the pre-surgical volumes of perifocal ^18^F-FDG PET and ^11^C-FMZ PET abnormalities in the VOI_A+H+PH_ and VOI_TL_, the resected abnormality volumes and the percentage of non-resected abnormality volumes were not significant and had a low prediction for Engel class I outcome for each individual patient as assessed by estimation of the C-statistic (Tables 6 and 7).

**Table 6:** Odds ratios from logistic regression of the pre-surgical volumes of perifocal ^18^F-FDG- and ^11^C-FMZ PET abnormalities in the VOI_A+H+PH_ , resected volumes of perifocal PET abnormalities, percentages of non-resected perifocal PET abnormalities and the MRI-derived mean resected volume and percentage this volume represents on probability of Engel class I outcome

^18^F-FDG-and ^11^C-FMZ PET, and MRI in VOI_A+H+PH_	Odds ratio	95 % CI	*p* value	C-statistic area under ROC curve
^18^F-FDG pre-surgical volume	1.0001	0.9995–1.0007	0.731	0.49
^18^F-FDG resected volume	1.0002	0.9991–1.0015	0.639	0.43
^18^F-FDG percentage of non-resected abnormality	1.0051	0.9446–1.0696	0.870	0.56
^11^C-FMZ pre-surgical volume	1.0007	0.9996–1.0018	0.204	0.62
^11^C-FMZ resected volume	1.0000	0.9978–1.0018	0.914	0.56
^11^C-FMZ percentage of non-resected abnormality	1.0229	0.9540–1.0968	0.524	0.63
MRI-derived volume resected	1.0000	0.9993–1.0007	0.901	0.45
MRI percentage of resected volume	0.9912	0.9338–1.0522	0.773	0.60

*VOI* volume of interest, _*A+H+PH*_ Amygdala+Hippocampus+Parahippocampal gyrus

**Table 7:** Odds ratios from logistic regression of the pre-surgical volumes of perifocal ^18^F-FDG- and ^11^C-FMZ PET abnormalities in the VOI_TL_ , resected volumes of perifocal PET abnormalities, percentages of non-resected perifocal PET abnormalities and the MRI-derived mean resected volume and percentage this volume represents on probability of Engel class I outcome

^18^F-FDG- and ^11^C-FMZ PET and MRI in VOI_TL_	Odds ratio	95 % CI	*p* value	C-statistic area under ROC curve
^18^F-FDG pre-surgical volume	1.0001	0.9999–1.0003	0.063	0.74
^18^F-FDG resected volume	1.0000	0.9999–1.0002	0.340	0.64
^18^F-FDG percentage of non-resected abnormality	1.0140	0.9412–1.0923	0.714	0.54
^11^C-FMZ pre-surgical volume	1.0001	0.9999–1.0003	0.259	0.60
^11^C-FMZ resected volume	1.0000	0.9997–1.0002	0.855	0.59
^11^C-FMZ percentage of non-resected abnormality	1.0281	0.9499–1.1127	0.491	0.55
MRI-derived volume resected	1.0000	0.9998–1.0002	0.500	0.61
MRI percentage of resected volume	1.0041	0.9033–1.1162	0.939	0.52

*VOI* volume of interest, _*TL*_ whole temporal lobe

### The results of the automated quantitative analysis of the MRI-derived resected volume

There was a marked difference between the mean volume of tissue resected in the VOI_A+H+PH_ between the right and left sides (2226 ± 1328 mm^3^ vs. 1187 ± 913 mm^3^, *p* = 0.006) and in the mean volume of tissue resected in the VOI_TL_ when comparing the right and left side (22,136 ± 7344 mm^3^ vs. 17,568 ± 4392 mm^3^,* p* = 0.020).

The post-surgical seizure outcome subgroups were not influenced by the volume of the MRI-derived resected brain tissue and the percentage this resected volume represented in the VOI_A+H+PH_ and VOI_TL_ (Tables 4 and 5). The odds ratios from logistic regression of the volume of MRI-derived resected brain tissue and the percentage the resected volume represented in the VOI_A+H+PH_ and VOI_TL_ were not significant and had a low prediction for Engel class I outcome for each individual patient as assessed by estimation of the C-statistic (Tables 6 and 7).

## Discussion

Anterior temporal lobectomy (ATL) has been established as an effective treatment option for patients with drug-resistant TLE, leading, in most reported series, to Engel class I seizure outcome (free of disabling seizures) in 60–80 % of patients [2, 4, 8, 12, 34, 35, 54]. The post-surgical seizure outcomes here are comparable with the data in the literature.

According to our best knowledge, this study is the first that examined by the automated quantitative voxel-based analysis whether the pre-surgical volume of perifocal ^18^F-FDG- and ^11^C-FMZ PET abnormalities in the VOI_A+H+PH_ and VOI_TL_, the extent of resection of the region of these abnormalities, and the percentage of the non-resected perifocal abnormality volume affected the post-surgical seizure outcomes. Additionally, we examined by the automated quantitative voxel-based analysis whether the MRI-derived resected volume of brain tissue of both VOIs influenced the post-surgical seizure outcome. A relatively small group of patients in this study is the most important limitation.

The most commonly used cerebral PET tracer in TLE is ^18^F-FDG, which measures glucose metabolism related to the synaptic and neuronal activity of the brain tissue [37]. Another cerebral PET tracer used to detect the epileptic brain region in patients with TLE is ^11^C-FMZ, which binds to α subunits of the γ-aminobutyric acid A benzodiazepine receptor. Decreased ^11^C-FMZ binding on interictal PET scans usually represents neuronal loss or receptor changes related to epileptogenicity [6, 9, 23, 31]. The majority of patients with refractory TLE exhibit on pre-operative ^18^F-FDG- and ^11^C-FMZ PET scans perifocal abnormalities of epileptogenic temporal lobe that may affect the surgical hypothesis. The pre-surgical evaluation in this study revealed perifocal ^18^F-FDG hypometabolism in 97 % patients, which is consistent with the published sensitivity of 85–96 % [5, 32, 45] and decreased ^11^C-FMZ binding in 81 % patients, which is also comparable with reported sensitivity of 85–100 % for detecting the epileptogenic cortex in patients with refractory TLE [19, 26, 27]. However, the automated quantitative evaluation in this study showed superior findings and revealed perifocal ^18^F-FDG- and ^11^C-FMZ abnormalities in all patients (100 %).

### The findings of the automated quantitative ^18^F-FDG- and ^11^C-FMZ PET evaluation

There is growing evidence that interictal ^18^F-FDG- and ^11^C-FMZ PET may represent functional seizure-related phenomena in the underlying temporal lobe and the functionally associated remote ipsilateral and contralateral regions [3, 6, 7, 16, 20, 21, 27, 32, 39, 41, 46, 48, 50–52] suggesting that the pre-surgical glucose hypometabolism and decreased flumazenil-binding pattern may be dynamic, seizure-related, and reversible. In the light of this evidence, the findings in the current series of cases are not surprising. We found that the pre-surgical volume of perifocal ^18^F-FDG hypometabolism and decreased ^11^C-FMZ binding in both VOIs did not influence post-surgical seizure outcome (Tables 4 and 5) and had low predictive values for the achievement of an Engel class I outcome for each individual patient with refractory TLE (Tables 6 and 7). These findings in both VOIs of the temporal lobe are consistent with the hypothesis that the area of perifocal ^18^F-FDG hypometabolism and decreased ^11^C-FMZ binding often indicate functionally impaired areas close to and often larger than the seizure focus but do not necessarily represent epileptogenic cortex [22, 23].

Following resection, the pre-surgical volume of the perifocal ^18^F-FDG- and ^11^C-FMZ PET abnormality in both VOIs of the temporal lobe was considerably reduced (Tables 2 and 3). However, the resected volumes of perifocal ^18^F-FDG- and ^11^C-FMZ PET abnormalities in both VOIs of the temporal lobe were relatively small and seizure outcomes were not affected by the amount of resected volume of perifocal abnormalities in either VOIs (Tables 4 and 5). Furthermore, the resected volumes had a low predictive value with regard to individual freedom from seizures (Tables 6 and 7). Additional support for this view is provided in this analysis demonstrating no substantial differences between the outcome subgroups in the proportion of the non-resected perifocal ^18^F-FDG hypometabolism and decreased ^11^C-FMZ binding in both VOIs (Tables 4 and 5), which also had a weak predictive value for individual freedom from seizures (Tables 6 and 7).

The volumetric findings of the current series of patients clearly suggest that complete resection of the pre-surgical perifocal ^18^F-FDG- and ^11^C-FMZ PET abnormality areas in the mesial structures of temporal lobe as well as in the entire temporal lobe is not a prerequisite for a satisfactory post-surgical seizure outcome in patients with drug-resistant TLE. However, it remains unclear why resection of only a small proportion of these abnormalities can result either in seizure freedom in the majority of patients or in unsatisfactory outcome in others.

Thus, our results support the idea that pre-surgical perifocal ^18^F-FDG- and ^11^C-FMZ PET abnormalities in both VOIs of the temporal lobe cannot be used to delineate the margin of surgical resection in these patients with TLE. Therefore, the suggestion of tailored resection delineated on the basis of the extent and severity of pre-surgical temporal lobe hypometabolism in patients with refractory TLE [53] seems questionable.

### The findings of the automated quantitative evaluation of the MRI-derived resected volume

The resection of epileptogenic tissue in the seizure onset temporal lobe is a prerequisite for achieving freedom from disabling seizures in patients with drug-resistant TLE. An element of the procedural discussions in TLE surgery is whether a reduced extent of resection is associated with a lower rate of seizure freedom and vice versa. One objective of this study was to assess the significance of the extent of resection of mesial structures (VOI_A+H+PH_) and of the whole temporal lobe (VOI_TL_), determined by automated quantitative analysis, to achieve seizure freedom after surgery for TLE. We compared the MRI-derived resected tissue volume of the VOI_A+H+PH_ and VOI_TL_ and the proportion of the resected tissue volume between the outcome subgroups and found no significant differences (Tables 4 and 5). Furthermore, the results show that the MRI-derived resected volume of the VOI_A+H+PH_ and VOI_TL_ as well as the proportion of this resected volume had a low predictive value for individual achievement of an Engel class I outcome after undergoing AMTL (Tables 6 and 7). These findings of the automated quantitative evaluation of the MRI-derived resected volume of the current series of patients are not a novelty and are consistent with the previous data in the literature, which showed that different surgical approaches in TLE and different amounts of mesial temporal resection do not result in different seizure outcomes [42, 43].

All patients with TLE in this cohort had on the pre-surgical MRI, ^18^F-FDG, and ^11^C-FMZ PET studies positive findings that corresponded with the epileptogenic temporal lobe as defined with multiple EEGs. Despite this fact, there were 22 % of patients who continued to experience seizures after surgery. It seems that the patients with TLE in this cohort do not have a uniform epilepsy condition and that performing a more or less “standardized” AMTL may not always be sufficient to remove all the tissue responsible for the occurrence of seizures. As noted earlier [35], ideally, every single patient with TLE should receive customized surgery to become seizure-free. However, to define the individualized resection margins more properly in patients with TLE, further diagnostic improvements are warranted.
